# A Kinesin Vdkin2 Required for Vacuole Formation, Mycelium Growth, and Penetration Structure Formation of *Verticillium dahliae*

**DOI:** 10.3390/jof8040391

**Published:** 2022-04-12

**Authors:** Xing Yang, Cuimei Guo, Chi Chen, Zhijuan Hu, Xinyao Zheng, Shan Xu, Xingyong Yang, Chengjian Xie

**Affiliations:** 1 The Chongqing Key Laboratory of Molecular Biology of Plant Environmental Adaptations, Chongqing Normal University, Chongqing 401331, China; 2019110513033@stu.cqnu.edu.cn (X.Y.); 2019110513007@stu.cqnu.edu.cn (C.G.); 2020110513007@stu.cqnu.edu.cn (C.C.); 2021110513003@stu.cqnu.edu.cn (Z.H.); 2021210513054@stu.cqnu.edu.cn (X.Z.); 20200017@cqnu.edu.cn (S.X.); 2 Chongqing Engineering Research Center of Specialty Crop Resources, The College of Life Science, Chongqing Normal University, Chongqing 401331, China; 3 College of Pharmacy, Chengdu University, Chengdu 610106, China; yangxingyong@cdu.edu.cn

**Keywords:** *Verticillium dahliae*, kinesin 2, vacuole, penetration peg, virulence

## Abstract

The soil-borne vascular fungus *Verticillium dahliae* infects hundreds of dicotyledonous plants, causing severe wilt diseases. During the initial colonization, *V. dahliae* develops a penetration peg to enable infection of cotton roots. In some phytopathogenic fungi, vacuoles play a critical role in normal formation of the infection structure. Kinesin 2 protein is associated with vacuole formation in *Ustilago maydis*. To identify the function of vacuoles in the *V. dahliae* infection structure, we identified *VdKin2*, an ortholog of kinesin 2, in *V. dahliae* and investigated its function through gene knockout. *VdKin2* mutants showed severe defects in virulence and were suppressed during initial infection and root colonization based on observation of green fluorescent protein-labeled *V. dahliae*. We also found that deletion of *VdKin2* compromised penetration peg formation and the derived septin neck. Disruption strains were viable and showed normal microsclerotia formation, whereas mycelium growth and conidial production were reduced, with shorter and more branched hyphae. Furthermore, the *VdKin2* mutant, unlike wild-type *V. dahliae*, lacked a large basal vacuole, accompanied by a failure to generate concentrated lipid droplets. Taken together, *VdKin2* regulates vacuole formation by *V. dahliae*, which is required for conidiation, mycelium growth, and penetration structure formation during initial plant root infection.

## 1. Introduction

*Verticillium dahliae*, the causative agent of *Verticillium* wilt disease, is a soil-borne pathogen that can infect over 200 plant species, including the economically important crop, cotton [1]. It is very difficult to control *V. dahliae* because of its colonization in the xylem and the persistence of resting structure microsclerotia [2]. Previous research indicated that *V. dahliae* develops a distinctive penetration peg from the hyphopodium for breaching the plant roots during initial colonization [3], and the penetration peg further develops a septin-organized hyphal neck to partition the hyphopodium and invasive hypha and forms a special fungus-host interface for secreting virulence effectors [4]. Effector VdSCP41 is delivered into the nucleus of plant cells through the fungus-host interface where it directly targets SARD1 and CBP60g, two key transcriptional factors, to inhibit plant immunity [5]. Similarly, the rice blast pathogen *Magnaporthe oryzae* forms a specialized infection structure called the appressorium on the plant leaf with which it infects its host [6]. The infection structure exerts physical force to penetrate the plant surface or deploys series of enzymes in a focused manner to digest the cuticle and plant cell wall [7].

Vacuoles are ubiquitous in the hyphae of filamentous fungi and have a broad range of functions including regulation of growth and death, degradation and turnover of cellular constituents, storage of ions and metabolites, nutrient transport, and cellular homeostasis [8]. Vacuoles are associated with the virulence of few phytopathogenic fungi [9]. During the maturing appressorium of *M. grisea*, the vacuole plays the central role of the lytic system and causes lipid droplets to sink. The vacuole undergoes considerable expansion to assist appressorium formation and generates osmotically active metabolites with the aid of the degradation of stored lipids [10]. This process increases the turgor pressure in the appressorium to promote forced entry of the fungal penetration peg into the plant epidermis. The vacuole also contributes to autophagy, which is critical for the normal appressorium formation and pathogenicity of *M. oryzae* [11]. It has also been reported that in *Candida albicans*, P-type ATP enzyme [12] and calcium channel Yvc1 [13] on tonoplasts can affect hyphae formation or elongation, thus weakening the virulence of *C. albicans*. However, the precise relationship between vacuoles and penetration pegs in *V. dahliae* is not well-understood. One of the main objectives of this study was to determine the function of the vacuole and explore its role in penetration and pathogenicity.

During the early stages of appressorium formation by *Colletotrichum lagenarium*, microtubules were observed to be arranged randomly in appressoria [14], whereas *M. oryzae* developed vertically arranged microtubule arrays that were unique to mature appressoria [15]. MST12, a homolog of the *Saccharomyces cerevisiae* STE12 transcription factor activated by MAPK pathways, is indispensable for the penetration peg and infectious hyphae growth in *M. grisea* [16], and the *mst12* mutant showed defects in microtubule reorganization related to penetration peg formation [15]. Fungal kinesins, generally considered to be microtubule-dependent motor proteins, play a role in the intracellular transport of secretory vesicles [17]. The microtubule-dependent motor enzyme Kin2, rather than Kin1, participates in the organization and formation of vacuoles in the plant pathogen *Ustilago maydis* [18], and deletion of Kin2 affects its pathogenicity [19]. Therefore, we identified the Kin2 orthologs and deleted the gene encoding this protein in *V. dahliae* to further investigate the function of the vacuoles.

## 2. Materials and Methods

### 2.1. Fungal Strains and Cultivation

The wild type of *V. dahliae* used v991 preserved in our laboratory. All the strains of *V. dahliae* used in this experiment were cultured in solid or liquid potato glucose medium in a constant temperature incubator at 25 °C. In this experiment, two mutants were named *ΔVdKin2-19* and *ΔVdKin2-43*, and a complementary strain was named *ΔVdKin2comp*. The aforementioned three strains were all constructed by our laboratory, and the wild type is referred to as WT.

### 2.2. Vector Construction and Fungal Transformation

To generate gene-replacement plasmid, two homologous arms were amplified with primer gene-5F/5R and gene-3F/3R from the genomic DNA of *V. dahliae* strain v991. The hygromycin B phosphotransferase resistance gene cassette (*Hyg^r^*) was amplified from the vector psilent1 (Hyg-F/R), and the three fragments were linked to *Xba* I/*Bam*H I linearized pGFP plasmid using the ligation-independent cloning method [20]. Transformants were selected with cefotaxime at 500 μg/mL and hygromycin B at 50 μg/mL. The gene’s deleted strains were screened according to our published protocol [20] and experimental materials and methods are detailed in Supporting Information Methods. To generate the gene complementation vector, the entire gene with its native promoter (1500 bp) and terminator (1000 bp) sequences (gene-com f/r) was cloned into the complementation plasmid pG418 [20] and introduced into the respective gene deletion strains *ΔVdKin2-19*. The screening of complementation strains was performed by detecting the expression of the gene using the RT-PCR method. Transformants were selected with cefotaxime at 500 µg/mL, hygromycin B at 50 µg/mL, and G418 at 25 µg/mL. To construct the VdSep5-GFP expression strains, VdSep5 was amplified from the cDNA of v991 and fused into *Xba* I/*Eco*R I-linearized pG418-pgad-GFP-TrpC and the vector was introduced into the *ΔVdKin2-19* or WT strains. Transformants were selected with cefotaxime at 500 µg/mL, hygromycin B at 50 µg/mL, and G418 at 25 µg/mL. All vectors were transformed into *V. dahliae* v991 via *Agrobacterium tumefaciens* AGL1 as described previously [20].

### 2.3. Pathogenicity Analysis

To evaluate the role of genes in the pathogenicity of *V. dahliae*, four-week-old *Gossypium hirsutum* were inoculated with spores (1 × 10^7^ spores/mL) from WT, *ΔVdKin2-19*, *ΔVdKin2-43*, *ΔVdKin2comp*, and sterile water (mock) using the root dip inoculation method as described previously [20]. Plants were grown in a greenhouse at 25 °C under a 16:8 h light-dark cycle [21]. Results were recorded approximately 20 days after inoculation with *V. dahliae*. To determine the colonization of *V. dahliae*, sections of cotton stems at the height of the first internode were collected, sterilized, placed on PDA medium with 50 μg/mL chloramphenicol, and incubated at 25 °C for one week [22]. All pathogenicity assays were independently performed three times with three plants for each genotype.

### 2.4. Root Colonization Analysis

Cotton plants were cultured in 1/2MS medium for two weeks to observe the pathogenic processes. The roots of 2-week-old cotton were immersed in a suspension of *V. dahliae* spores (1 × 10^5^ spores/mL) from WT/eGFP and *ΔVdKin2*/eGFP strains for 10 min. Thereafter, the shoots were gently washed with sterilized water and replanted in the 1/2MS medium. After 48 h of treatment, the roots were placed on a slide with a drop of distilled water and then covered with a cover slip for microscopic examination. Fluorescent photographs were taken using a confocal laser microscope under 40× objective lenses (LSM900, Carl Zeiss, Oberkochen, Germany). During microscopy, a 488 nm laser was used to excite GFP.

### 2.5. Salt-Stress Response

For abiotic stress assays, PDA plates were supplemented with KCl (0.7 M) or sorbitol (1 M). PDA plates without such supplements were used as controls. Drops of spore suspensions were cultured on PDA plates with or without supplements and incubated at 25 °C. The colony diameters of all strains were measured after 10 days of incubation. Relative growth inhibition (RGI) assessed stress tolerance, RGI = (D_C_ − D_T_)/D_C_ × 100%, D_C_, and D_T_ indicated the diameter of the colony on the control and stress plates, respectively [23]. All experiments were repeated thrice.

### 2.6. Microsclerotial Formation Assays

To determine whether *VdKin2* is required for microsclerotial formation, all strains (WT, *ΔVdKin2-19*, and *ΔVdKin2-43* or *VdKin2comp*) were inoculated on the nitrocellulose membrane, which was overlaid on solid microsclerotia-inducing medium (glucose, 5.0 g/L; NaNO_3_, 0.2 g/L; KCl, 0.52 g/L; MgSO_4_∙7H_2_O, 0.52 g/L; K_2_HPO_4_, 1.52 g/L; vitamin B1, 3.0 μM; vitamin H, 0.1 μM; agar, 15 g/L). After 15 days, hyphal and microsclerotial samples were collected for microscopic observation.

### 2.7. Penetration Assays

The sterilized cellophane membrane was placed on the minimal medium. Thereafter, a 5 μL suspension of conidia (1 × 10^7^ spores/mL) was loaded and kept at 25 °C for 3 days. After the cellophane membrane was removed, the culture was continued for 2 days [4]. The cellophane membrane with colonies was placed on a glass slide, and the remaining hyphae, hyphopodia, septin ring, and penetration peg were observed under an optical microscope (DM3000, Carl Zeiss, Oberkochen, Germany). All experiments were repeated three times.

### 2.8. Hyphal Septa, Vacuoles, and Lipid Droplet Staining

The WT, *ΔVdKin2*, and *ΔVdKin2comp* strains were grown on solid PDA medium for 5 days at 25 °C. Mycelium and spores were collected and stained using the following methods.

To observe the vacuoles, a stock solution of FM4-64 (1 mg/mL in DMSO) was prepared, diluted in distilled H_2_O at a final concentration of 5 μg/mL, and kept on ice. Mycelium and spores were washed with PBS buffer, and about 20 μL working solution was dropped on the glass slide and left to stain for 30 min at room temperature. Before observation, the mycelium and spores were washed with PBS buffer.

To observe the septa, the mycelium was washed with sterile water, and then the mycelium was strained with freshly prepared Calcofluor white (CFW) dye with a working concentration of 1 mg/mL, strained for 10 min at 25 °C in dark, and washed with PBS buffer.

To observe the lipid droplets, spores were washed with sterile water. Nile red strain solution (SL0201, Coolaber, Beijing, China) was diluted with distilled H_2_O to a working concentration of 0.1 mg/mL, dyed for 10 min at 25 °C in the dark, and washed with PBS buffer. Lipid droplets were visualized using a spinning disk confocal microscope using an excitation filter with a range of 485–543 nm. The red fluorescence signal was observed using a fluorescence microscope under 63× objective lenses (DM3000, Carl Zeiss, Oberkochen, Germany).

### 2.9. RNA-Seq

All strains (WT and Δ*VdKin2*) used for transcriptome analyses were inoculated on a cellophane membrane overlaid on potato dextrose agar medium for 10 days. Total RNA was extracted using TRIzol reagent (Invitrogen Life Technologies, Waltham, MA, USA). Before transcriptome sequencing, the concentration, quality, and integrity were determined using a NanoDrop spectrophotometer (Thermo Scientific, Waltham, MA, USA). RNA (3 µg) was used as the input material for the RNA sample preparations. Sequencing libraries were generated according to the following steps. First, mRNA was purified from total RNA using poly T oligo-attached magnetic beads. Fragmentation was carried out using divalent cations at elevated temperatures in an Illumina proprietary fragmentation buffer. First-strand cDNA was synthesized using random oligonucleotides and SuperScript II. Second-strand cDNA synthesis was subsequently performed using DNA Polymerase I and RNase H. Remaining overhangs were converted into blunt ends via exonuclease/polymerase activities, and the enzymes were removed. After adenylation of the 3ʹ ends of the DNA fragments, Illumina PE adapter oligonucleotides were ligated to prepare for hybridization. To select cDNA fragments of the preferred 400–500 bp in length, the library fragments were purified using the AMPure XP system (Beckman Coulter, Beverly, CA, USA). DNA fragments with ligated adaptor molecules on both ends were selectively enriched using the Illumina PCR Primer Cocktail in a 15-cycle PCR reaction. The products were purified (AMPure XP system) and quantified using an Agilent high-sensitivity DNA assay on a Bioanalyzer 2100 system (Agilent, Santa Clara, CA, USA). The sequencing library was then sequenced on the NovaSeq 6000 platform (Illumina, San Diego, CA, USA) by Shanghai Personal Biotechnology Cp. Ltd., Shanghai, China. Clean reads filtered from the raw transcriptome sequence data were mapped to the reference genome of *V. dahliae*, and these mapped reads were used for further analyses.

### 2.10. RT-PCR Assay

Total RNA was isolated using the OminiPlant RNA Kit (CWBIO, Cambridge, MA, USA). Isolated total RNA was reverse-transcribed using the PrimeScript RT Reagent Kit (TaKaRa Dalian Biotechnology, Dalian, China). Expression of *VdKin2* was determined by RT-PCR. PCR cycling consisted of an initial step of denaturation at 94 °C for 2 min, followed by 25 cycles of 94 °C for 30 s, 57 °C for 30 s, and 72 °C for 30 s. The 18sRNA gene of *V. dahliae* was used as a reference control. All primers used in this study are listed in Supplementary Table S1. The *V. dahliae* v991 transformants used in this study are listed in Supplementary Table S2.

## 3. Results

### 3.1. Identification of Vdkin2

**Figure 1:**
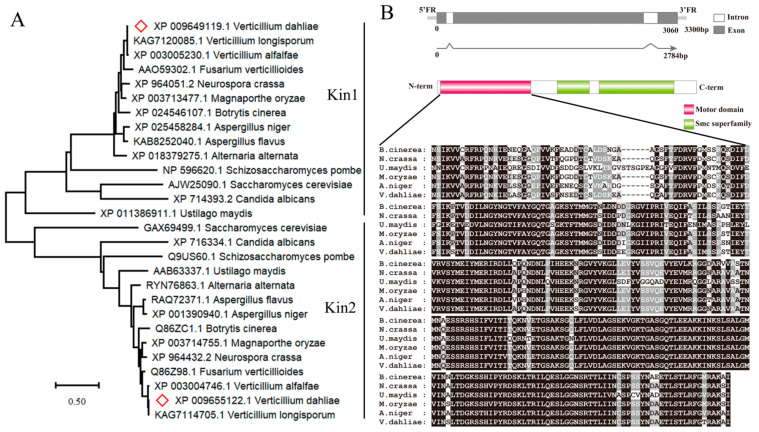
Sequence analysis of VdKin2. (**A**) Phylogenetic tree of VdKin1 and VdKin2 in *Verticillium dahliae* v991 and their homologs from the annotated NCBI protein database in other fungi. The phylogenetic tree was constructed with the maximum likelihood method with MEGA X software. The protein evolutionary model was analyzed using the “find best protein model”, resulting in LG + G + I, and bootstrap values were based on 1000 replicates. (**B**) Genomic and cDNA of VdKin2 were compared using MultAlin. Gray and white represent exons and introns, respectively. Protein domains of VdKin2 were predicted using the NCBI Conserved Domain Database, which contain a motor domain and Smc superfamily domain (chromosome segregation ATPase). Multiple sequence alignments of the motor domains of *Botrytis cinerea*, *Neurospora crassa*, *Ustilago maydis*, *Magnaporthe oryzae*, *Aspergillus niger* and *V. dahliae* are included.

Phylogenetic analysis suggested that fungal motor enzyme genes were divided into two subfamilies: Kin1-type and Kin2-type (Figure 1A). In the *V. dahliae* genome, a single-copy Kin2-type gene (VDAG_09024) was identified as homologous to *U. maydis* Kin2 (54.05% overall identity). Here, VDAG_09024 was designated as VdKin2. The *VdKin2* sequence includes three exons and two introns with an open reading frame of 928 codons, and the deduced molecular weight of VdKin2 was 102.6 kDa (Figure 1B). The VdKin2 sequence harbors a motor domain and Smc superfamily domain (chromosome segregation ATPase). Multiple sequence alignment confirmed that the kinesin motor domains of Kin2 orthologs from different fungi are highly conserved (Figure 1B).

### 3.2. Vdkin2 Is Required for Virulence in Cotton

**Figure 2:**
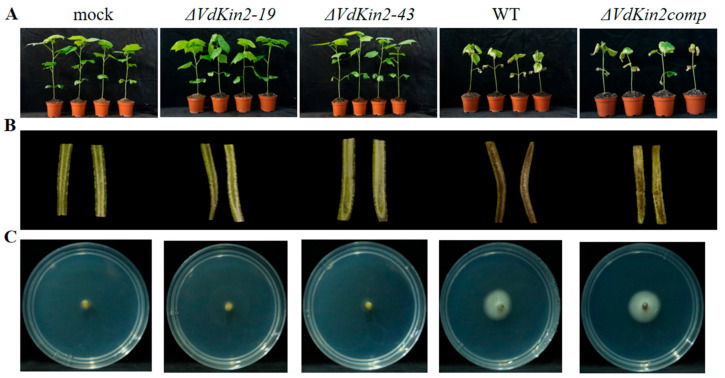
*ΔVdKin2* strains exhibit severe defects in pathogenicity. (**A**) Four-week-old cotton seedlings were inoculated with wild-type (WT), *ΔVdKin2-19*, *ΔVdKin2-43*, *ΔVdKin2comp*, or sterile water (mock) at 10^7^ spores/mL and cultured in a greenhouse. Symptoms of *Verticillium* wilt were photographed at 20 days after inoculation. (**B**) Discoloration of the inoculation shoot longitudinal sections. (**C**) Fungal outgrowth after one week of plating stem sections. The experiments were repeated 3 times.

To determine the role of VdKin2 in virulence, we generated gene-deletion strains using homologous recombination (Supporting Information Figure S1) and then reintroduced the wild-type gene into the corresponding target gene-deletion strains. Pathogenicity assays were performed on cotton (*G. hirsutum*) seedlings by root dip inoculation of the *VdKin2* deletion mutant, complemented mutant, and wild-type strains. At 20 days post-inoculation, severe leaf chlorosis was observed in plants infected with the wild-type (WT) and *ΔVdKin2comp* strains (Figure 2A and Figure S2), which was accompanied by discoloration of the vascular system (Figure 2B). In contrast, plants infected with the *VdKin2* mutant did not develop symptoms and were indistinguishable from uninfected plants (Figure 2A). Correspondingly, *V. dahliae* could only be successfully re-isolated from the hypocotyls of plants infected with the WT and *ΔVdKin2comp* strains (Figure 2C).

### 3.3. Deletion of Vdkin2 Impairs Penetration of V. dahliae

**Figure 3:**
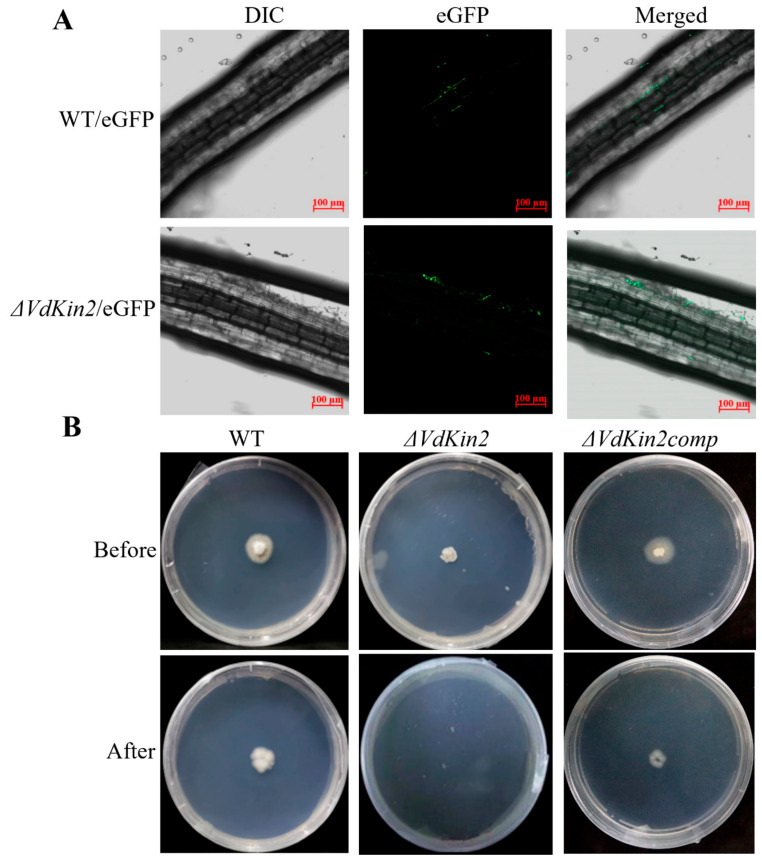
Root colonization and penetration analyses of *Verticillium dahliae*. (**A**) Roots of 2-week-old cotton were inoculated with wild-type (WT)/eGFP and *ΔVdKin2*/eGFP strains at 10^5^ spores/mL for 48 h to observe invasion and colonization. (**B**) Cellophane membranes were plated onto minimal medium and inoculated with conidia of WT, *ΔVdKin2*, and *ΔVdKin2comp* strains at 25 °C for 3 days, the cellophane was removed, and culture was continued for 2 days. The top panels show colonies of the strain grown on the cellophane membranes. The bottom panels show colonies grown from hyphae penetrating the membrane after removing the cellophane membrane.

The roots of host plants are entry points for *V. dahliae*, and thus the roots of 2-week-old cotton plants were infected with enhanced green fluorescent protein (eGFP)-expressing strains to evaluate the differences in root entry between the WT and *ΔVdKin2* mutants. After 48 h, the WT/eGFP strain was detected in the vascular bundles of cotton. In contrast, *ΔVdKin2*/eGFP was only observed in the peripheral region of the roots but not in the xylem vessels (Figure 3A). To further confirm the effect of *ΔVdKin2* knockout on root colonization, WT, *ΔVdKin2*, and *ΔVdKin2comp* strains were inoculated on cellophane membranes that had been laid on minimal medium. The *ΔVdKin2* strain exhibited obvious defects in the ability of hyphae to penetrate the cellophane membrane, whereas the WT and *ΔVdKin2comp* strains successfully penetrated the cellophane membrane, and fungal growth on the medium was observed when the cellophane membrane was removed after 2 days (Figure 3B). These results indicate that deletion of *VdKin2* impaired *V. dahliae* colonization in the vascular bundles of cotton.

**Figure 4:**
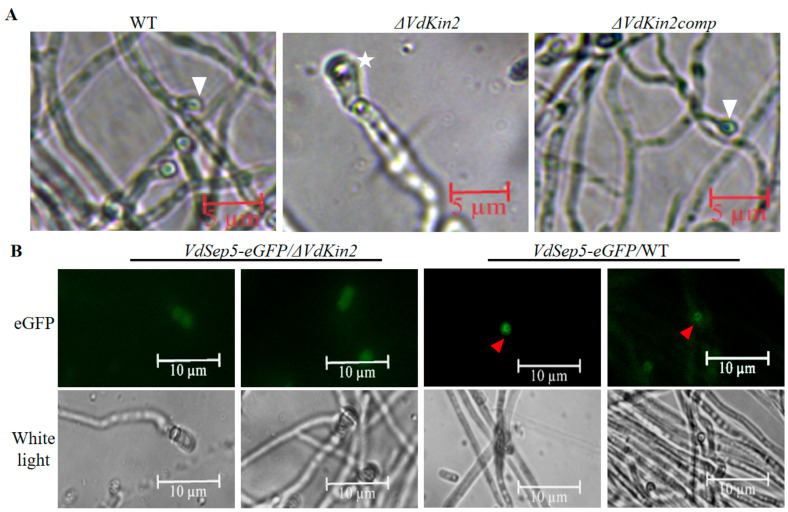
*VdKin2* is indispensable for penetration peg and septin ring formation in *Verticillium dahliae*. (**A**) Penetration pegs formed on the cellophane membrane by wild type (WT), *ΔVdKin2*, or *ΔVdKin2comp* strains. White arrows indicate penetration pegs. The *ΔVdKin2* strain failed to form penetration pegs (white star). (**B**) Cellular localization of VdSep5-GFP in WT and *ΔVdKin2* during development of the penetration peg. The red arrow indicates the septin ring.

A previous study showed that *V. dahliae* forms a penetration peg and derived septin neck to pierce plant roots. Therefore, we first observed hyphae on the cellophane membrane after 3 days of inoculation under microscopy. The *ΔVdKin2* strain developed defective hyphopodia that were incapable of producing penetration pegs, whereas the WT and complemented strains produced penetration pegs on the cellophane membrane (Figure 4A). VdSep5-GFP was further expressed in the WT and *ΔVdKin2* strains and a compact septin ring signal was observed, whereas there was no clear ring signal in the *ΔVdKin2* strains (Figure 4B). Thus, deletion of *VdKin2* impairs formation of the penetration structure and ultimately compromises *V. dahliae* colonization in the vascular bundles in the xylem of cotton.

### 3.4. VdKin2 Deletion Leads to Aberrant Hyphal Growth, Reduced Conidiation, and Abnormal Vacuole Morphology

**Figure 5:**
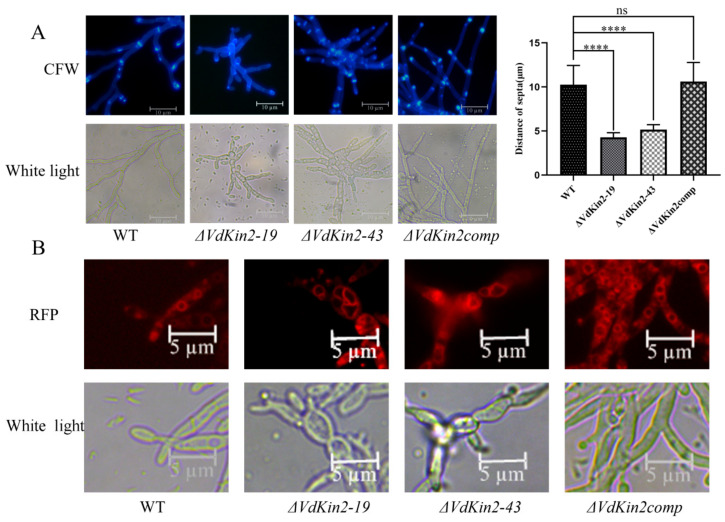
VdKin2 affects septa development and vacuole morphology in *Verticillium dahliae*. (**A**) Hyphae of wild type (WT), *ΔVdKin2*, or *ΔVdKin2comp* strains were stained with Calcofluor White and the septa distance was observed. Scale bar = 10 μm. Measurement of septa using ImageJ software. The error bars represent standard deviations. Asterisks (****) indicate significance at *p* < 0.001. The experiments were performed three times independently. (**B**) Vacuoles were dyed with FM4-64 and photographs were acquired by fluorescence microscopy with DIC and RFP filters. Scale bar = 5 μm.

Deletion of *VdKin2* led to aberrant hyphal growth, and *VdKin2* colonies showed slow growth and an abnormal shape. The cell walls and septa were stained with Calcofluor White and observed by fluorescence microscopy after growth in potato dextrose agar medium. The results indicated that the *ΔVdKin2* strains had more branching hyphae than the WT and complemented strains, and the distance between septa in the *ΔVdKin2* strains was less than that in the WT and complemented strains in elongated hyphae (Figure 5A).

Kin2 plays an important role in vacuole organization and formation. Vacuoles in hyphae were stained with FM4-64 and observed under a fluorescence microscope. Vacuoles in the WT and complemented strains were round and massive, whereas they had an irregular morphology in the *ΔVdKin2* strains (Figure 5B). Thus, VdKin2 is necessary for hyphal branching, septa positioning, and vacuole morphology.

**Figure 6:**
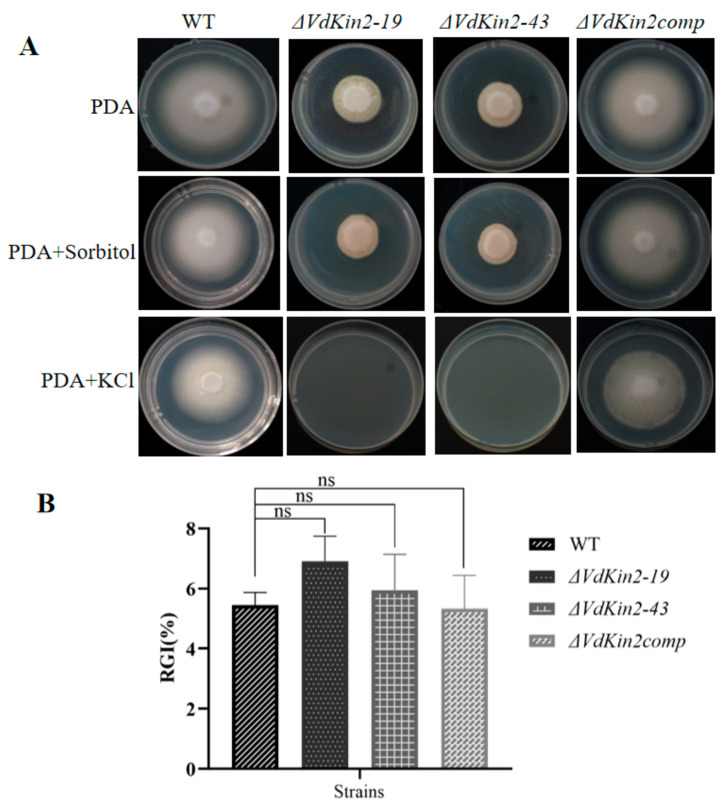
*VdKin2* is required for high-salt stress resistance of *Verticillium dahliae*. (**A**) Colony morphology of wild type (WT), *ΔVdKin2*, and *ΔVdKin2comp* strains after 10 days of growth on potato dextrose agar (PDA) medium containing 1 M sorbitol or 0.75 M KCl. (**B**) Relative inhibition rate of growth of *V. dahliae* cultivated in PDA medium plus 1 M sorbitol. The data are shown as the mean ± standard error of the mean. ns, not significant.

We also examined the sensitivity of *ΔVdKin2* strains to high-salt stress and high-osmolarity stress. Compared with the WT strain and complemented strain, the *ΔVdKin2* strains exhibited dramatic hypersensitivity to high-salt stress. Under high osmolarity stress, the relative growth inhibition value did not significantly differ between the WT and *ΔVdKin2* strains on high-osmolarity stress medium (Figure 6). Moreover, compared with that in the WT and complemented strains, conidia production in *ΔVdKin2* strains was significantly decreased (Figure S3). However, there was no significant effect on microsclerotia formation (Figure S4).

### 3.5. VdKin2 Deletion Affects Lipid Storage in the Vacuoles

**Figure 7:**
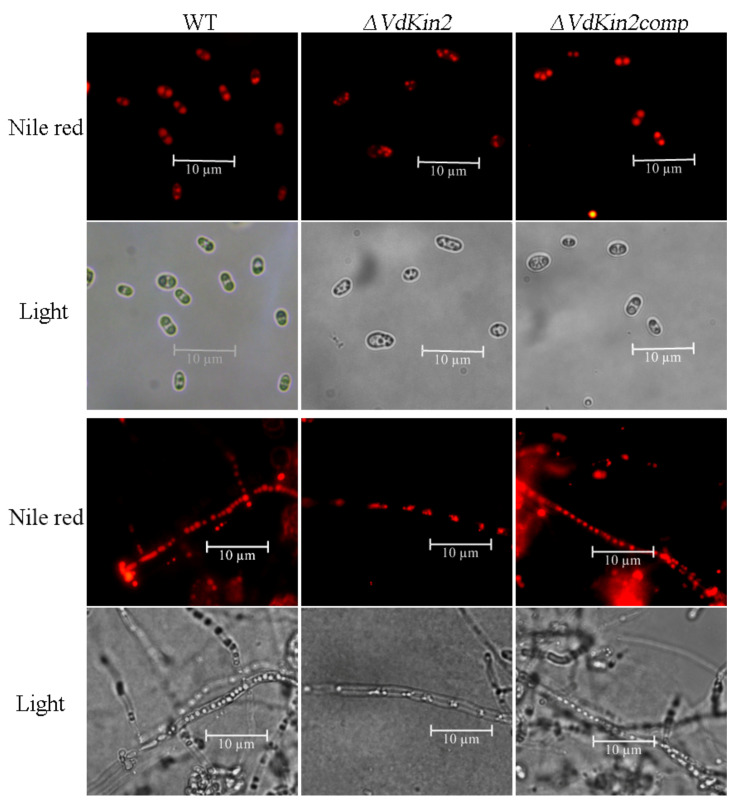
Deletion of *VdKin2* impairs lipid droplet development. Lipid droplets were stained using Nile red. Conidia and hyphae from the wild type (WT), *ΔVdKin2*, and *ΔVdKin2comp* strains.

Because the vacuole is a central element of the sinking of lipid droplets [10], lipid droplets were detected by staining with Nile red, a lipid-droplet-specific fluorescent dye. Notably, concentrated lipid droplets were observed in the WT and complemented strains, whereas only loose and small volumes of lipids were observed in the *ΔVdKin2* strains (Figure 7). These results indicate that deletion of *VdKin2* impairs the sinking of lipid droplets because of abnormal vacuole development.

### 3.6. RNA-Sequencing Analysis

To investigate the effect of *VdKin2* deletion on gene expression, transcriptomic analysis was conducted between the *VdKin2* mutants and WT strains. The strains were inoculated on a cellophane membrane overlaid on potato dextrose agar medium for 10 days (Figure S5), and then RNA samples were extracted for RNA-sequencing analysis.

**Figure 8:**
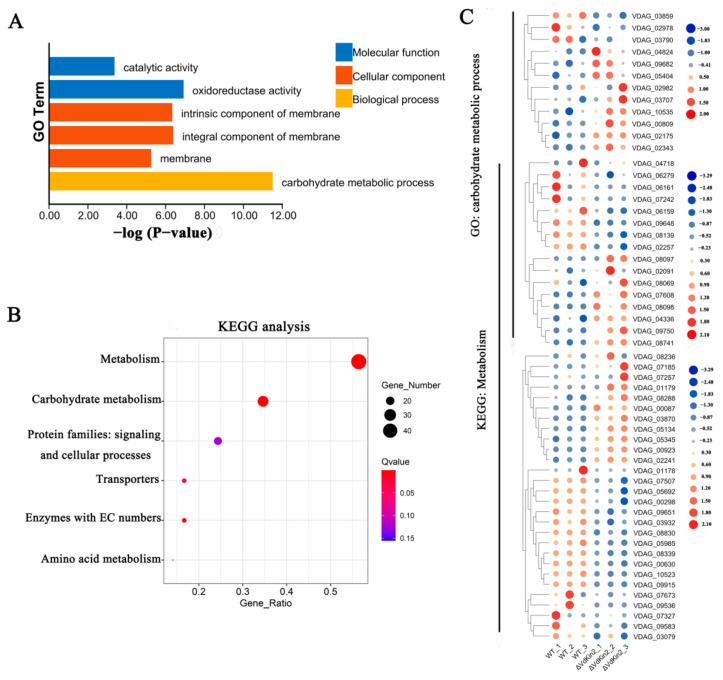
Gene Ontology (GO) annotation (**A**) and Kyoto Encyclopedia of Genes and Genomes (KEGG) enrichment analysis (**B**) for differentially expressed genes. GO enrichment (*p* < 0.05) and KEGG pathway enrichment (*p* < 0.05). (**C**) Heat maps of genes involved in “carbohydrate metabolic process” and “metabolism”. Log^2^ (FPKM) values are shown relative to the mean expression across all samples.

Statistical analysis revealed 484 differentially expressed genes (*p*-value < 0.01) between the *ΔVdKin2* and WT strains, among which 237 genes were upregulated and 247 genes were downregulated in *ΔVdKin2* strains. To characterize the functional processes of differentially expressed genes, these genes were analyzed to determine their Gene Ontology and Kyoto Encyclopedia of Genes and Genomes pathways. Among the differentially expressed genes, those involved in “carbohydrate metabolic processes” were significantly enriched. Kyoto Encyclopedia of Genes and Genomes cluster analysis showed that a high percentage of genes were involved in “metabolism”, with “carbohydrate metabolism” having a greater proportion in “metabolism” (Figure 8). These results indicate that Vdkin2 is involved in carbohydrate metabolism in *V. dahliae*.

## 4. Discussion

The vacuole has diverse functions in fungi; however, its function has not been clearly elucidated in the penetration structure formation of *V. dahliae*. *Kin2* encoding the heavy chain of conventional kinesin is involved in the formation of the vacuoles of *U. maydis*. For this reason, we attempted to explore the function of *Kin2* with respect to vacuole formation through functional analysis of *Kin2* in *V. dahliae*. Similarly to other filamentous fungi, the *V. dahliae* v991 genome contains only a single copy of *VdKin2.* Moreover, these kin2 proteins were located in the same branch of the evolutionary tree with a highly conserved functional domain.

To examine the function of VdKin2, we knocked out *VdKin2* in *V. dahliae* v991. Deletion of *VdKin2* severely affected hyphal growth, in agreement with Lehmler’s findings [19]. However, the mutants showed normal spore morphology and also produced a large quantity of spores, despite having fewer than the wild type. In the fission yeast *S. pombe*, a kinesin-like gene, *tea2p*, promotes microtubule growth, which is important for establishing and maintaining polarized growth along the long axis of the cell [24]. KipA, an orthologous protein of tea2p, is also required for normal microtubule organization and polarized growth of the filamentous fungus *Aspergillus nidulans* [25]. One of the most extremely polarized kinds of cells in nature is the indefinitely growing hypha of filamentous fungi [26]. However, not all kinesins have an essential role in hyphal growth [27]. The hyphal growth defect of the *VdKin2* mutant indicated that VdKin2 was indispensable for the polarized hyphal growth of *V. dahliae*. Kin2 is a microtubule-dependent motor enzyme which is involved in the formation of vacuoles [18], and thus we detected vacuoles in *V. dahliae*. As we predicted, the *ΔVdKin2* strains formed abnormal vacuole morphology.

Additionally, vacuoles are central elements of the lytic system and the sinking of lipid droplets in the maturing appressoria of *M. grisea*, which are crucial for the penetration of plant hard surfaces [28]; thus, we observed lipid droplets in *V. dahliae.* Concentrated lipid droplets were observed in the WT strain but not in the *ΔVdKin2* strains. Based on these data, our results add further support to the notion that the vacuole is indispensable for lipid droplet storage. Vacuole and lipid droplets were shown to play a critical role in the penetration structure and pathogenicity of *M. grisea* [11]. Consistently, the *ΔVdKin2* mutant showed the loss of virulence in cotton plants. Further penetration ability analysis revealed that the *ΔVdKin2* mutant cannot format penetration pegs and septin necks to penetrate the root epidermis or cellophane membrane. Our data support the essential role of *VdKin2* as a key component in normal vacuole function for penetration peg formation and septin ring organization in *V. dahliae.*

Unexpectedly, *ΔVdKin2* strains showed high salt sensitivity but were not sensitive to high osmotic pressure. Vacuoles act as storage and sequestration vessels for a wide variety of ions and are responsible for cytosolic ion homeostasis and responses to ionic shock [9]. Thus, the abnormal vacuole morphology of the *ΔVdKin2* strain may have impaired the function of ion transporters localized to the vacuole and the mutants were not able to maintain ion homoeostasis within cells. In summary, analyses of the *ΔVdKin2* strain revealed linkages among vacuoles, lipid droplets, a penetration structure, and fungal pathogenicity. However, more functions of vacuoles remain to be revealed.

## Supplementary Materials

The following supporting information can be downloaded at: https://www.mdpi.com/2309-608X/8/4/391/s1. Figure S1. *VdKin2* knockout and complementation in *V. dahliae*. Figure S2. The results of the pathogenic analysis. Figure S3. Number of conidia calculated in potato dextrose broth medium. Figure S4. Strains were cultured on solid microsclerotia-inducing medium to induce microsclerotial formation. Figure S5. Strains were cultured on solid microsclerotia-inducing medium with or without cellophane membrane. Table S1. Primers used in this study. Table S2. *V. dahliae* strains used in this study. Supporting Information Methods.
