# Correction: Yang et al. A Kinesin Vdkin2 Required for Vacuole Formation, Mycelium Growth, and Penetration Structure Formation of *Verticillium dahliae*. *J. Fungi* 2022, *8*, 391

**DOI:** 10.3390/jof8070707

**Published:** 2022-07-04

**Authors:** Xing Yang, Cuimei Guo, Chi Chen, Zhijuan Hu, Xinyao Zheng, Shan Xu, Xingyong Yang, Chengjian Xie

**Affiliations:** 1 The Chongqing Key Laboratory of Molecular Biology of Plant Environmental Adaptations, Chongqing Normal University, Chongqing 401331, China; 2019110513033@stu.cqnu.edu.cn (X.Y.); 2019110513007@stu.cqnu.edu.cn (C.G.); 2020110513007@stu.cqnu.edu.cn (C.C.); 2021110513003@stu.cqnu.edu.cn (Z.H.); 2021210513054@stu.cqnu.edu.cn (X.Z.); 20200017@cqnu.edu.cn (S.X.); 2 Chongqing Engineering Research Center of Specialty Crop Resources, The College of Life Science, Chongqing Normal University, Chongqing 401331, China; 3 College of Pharmacy, Chengdu University, Chengdu 610106, China; yangxingyong@cdu.edu.cn

## Figure Legend

In the original publication [1], there was a mistake in the legend for Figure 5. “The white arrow represents the septa” should be deleted. The correct legend appears below:

**Figure 5.** VdKin2 affects septa development and vacuole morphology in *Verticillium dahliae*. (**A**) Hyphae of wild type (WT), *ΔVdKin2*, or *ΔVdKin2comp* strains were stained with Calcofluor White and the septa distance was observed. Scale bar = 10 μm. Measurement of septa using ImageJ software. The error bars represent standard deviations. Asterisks (****) indicate significance at *p* < 0.001. The experiments were performed three times independently. (**B**) Vacuoles were dyed with FM4-64 and photographs were acquired by fluorescence microscopy with DIC and RFP filters. Scale bar = 5 μm.

## Text Correction

1. In Section *2.8. Hyphal Septa, Vacuoles, and Lipid Droplet Staining*, Paragraph 3, regarding the staining method of the septum, the “Congo red” should be replaced by “Calcofluor white (CFW)”. The correct paragraph appears below:

To observe the septa, the mycelium was washed with sterile water, and then the mycelium was strained with freshly prepared Calcofluor white (CFW) dye with a working concentration of 1 mg/mL, strained for 10 min at 25 °C in dark, and washed with PBS buffer.

2. In Section *3.1. Identification of Vdkin2*, Paragraph 1, “an open reading frame of 822 codons” should be modified to “an open reading frame of 928 codons”, and “86.6 kDa” should be “102.6 kDa”. The correct paragraph appears below:

Phylogenetic analysis suggested that fungal motor enzyme genes were divided into two subfamilies: Kin1-type and Kin2-type (Figure 1A). In the *V. dahliae* genome, a single-copy Kin2-type gene (VDAG_09024) was identified as homologous to *U. maydis* Kin2 (54.05% overall identity). Here, VDAG_09024 was designated as VdKin2. The *VdKin2* sequence includes three exons and two introns with an open reading frame of 928 codons, and the deduced molecular weight of VdKin2 was 102.6 kDa (Figure 1B). The VdKin2 sequence harbors a motor domain and Smc superfamily domain (chromosome segregation ATPase). Multiple sequence alignment confirmed that the kinesin motor domains of Kin2 orthologs from different fungi are highly conserved (Figure 1B).

The authors apologize for any inconvenience caused and state that the scientific conclusions are unaffected. This correction was approved by the Academic Editor. The original publication has also been updated.
