# Supplementary material for: A Kinesin Vdkin2 Required for Vacuole Formation, Mycelium Growth, and Penetration Structure Formation of Verticillium dahliae
Source: J Fungi (Basel). 2022 Apr 12;8(4):391. doi: 10.3390/jof8040391 (PMC9030024; doi:10.3390/jof8040391)
Supplement: Supplementary file 1 [file jof-08-00391-s001.zip › jof-1597028-supplementary.pdf]

Journal of Fungi

Supplementary Materials

A Kinesin Vdkin2 Required for Vacuole Formation, Mycelium Growth, and Penetration Structure Formation of *Verticillium dahliae*

Xing Yang [1,2], Cuimei Guo [1,2], Chi Chen [1,2], Zhijuan Hu [1,2], Xinyao Zheng [1,2], Shan Xu [1,2], Xingyong Yang [3] and Chengjian Xie [1,2,*]

[1] The Chongqing Key Laboratory of Molecular Biology of Plant Environmental Adaptations, Chongqing Normal University, Chongqing 401331, China; 2019110513033@stu.cqnu.edu.cn (X.Y.); 2019110513007@stu.cqnu.edu.cn (C.G.); 2020110513007@stu.cqnu.edu.cn (C.C.); 2021110513003@stu.cqnu.edu.cn (Z.H.); 2021210513054@stu.cqnu.edu.cn (X.Z.); 20200017@cqnu.edu.cn (S.X.)
[2] Chongqing Engineering Research Center of Specialty Crop Resources, The College of Life Science, Chongqing Normal University, Chongqing 401331, China
[3] College of Pharmacy, Chengdu University, Chengdu 610106, China; yangxingyong@cdu.edu.cn
* Correspondence: xcj614@163.com or 20131913@stu.cqnu.edu.cn; Tel.: +86-13594334787

ASSOCIATED CONTENT

Supplementary Figures and Tables

Figure S1. *VdKin2* knockout and complementation in *Verticillium dahliae*.

Figure S2. The results of the pathogenic analysis.

Figure S3. Number of conidia calculated in potato dextrose broth medium.

Figure S4. Strains were cultured on solid microsclerotia-inducing medium to induce microsclerotial formation.

Figure S5. Strains were cultured on solid microsclerotia-inducing medium with or without cellophane membrane.

Table S1. Primers used in this study.

Table S2. *Verticillium dahliae* strains used in this study.

Supporting Information Methods

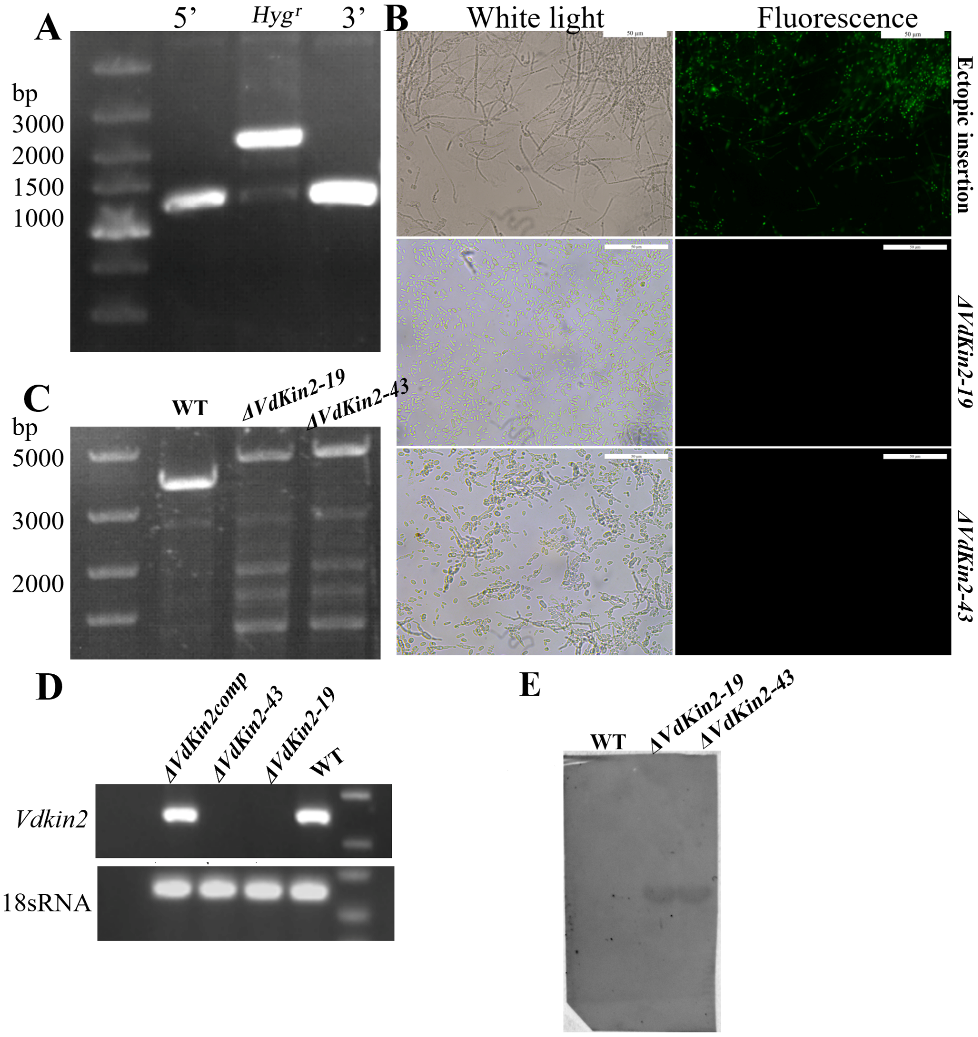

Figure S1. *VdKin2* knockout and complementation in *Verticillium dahliae*. (A) 5′- and 3′-flanking region of *VdKin2* and hygromycin resistance gene (*Hyg^r*). (B) GFP was not observed in null mutants but was present in ectopic insertion transformants or null mutants with ectopic insertions. (C) Gene-deletion event was verified using a pair of primers complementary to the region outside of the two homologous arms of the *VdAA9*-deletion cassettes. (D) RT-PCR revealed the elimination of *Vdkin2* in gene-deletion mutants. 18S rRNA served as an internal control. (E) Southern blotting indicates a single-copy insertion of the *Hyg^r* gene in *VdKin2* mutants.

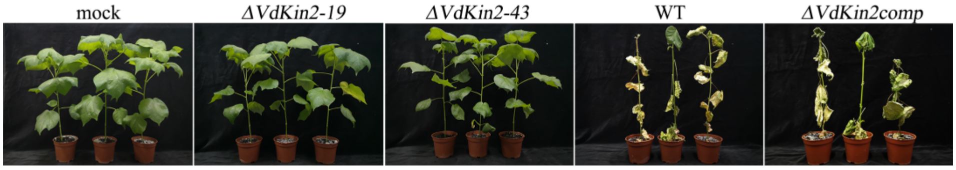

Figure S2. The results of the pathogenic analysis. Four-week-old cotton seedlings were inoculated with wild-type (WT), *ΔVdKin2-19*, *ΔVdKin2-43*, or *ΔVdKin2comp* or sterile water (mock) at 10^7 spores/mL and cultured in a greenhouse. Symptoms of *Verticillium* wilt were photographed at 40 days after inoculation.

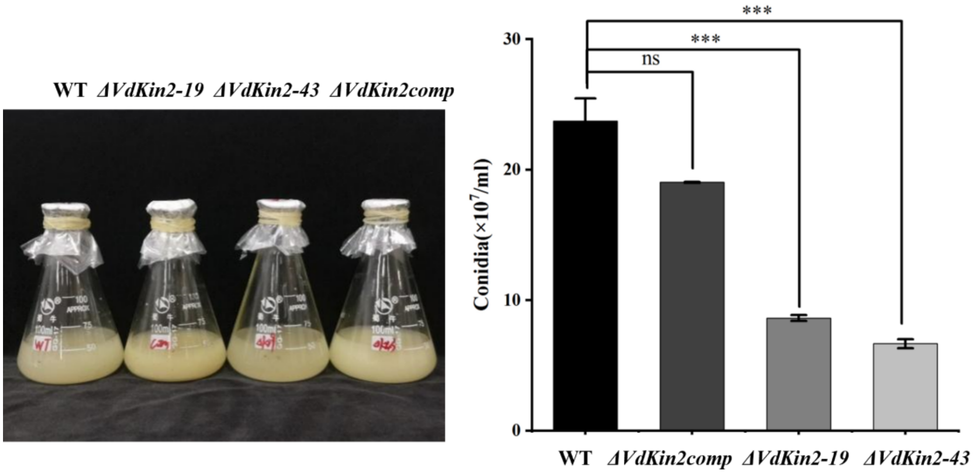

Figure S3. Number of conidia calculated in potato dextrose broth medium. Values are the means plus standard deviations (error bars) from three replicates. ***significant differences at P < 0.001, based on Student's *t*-test.

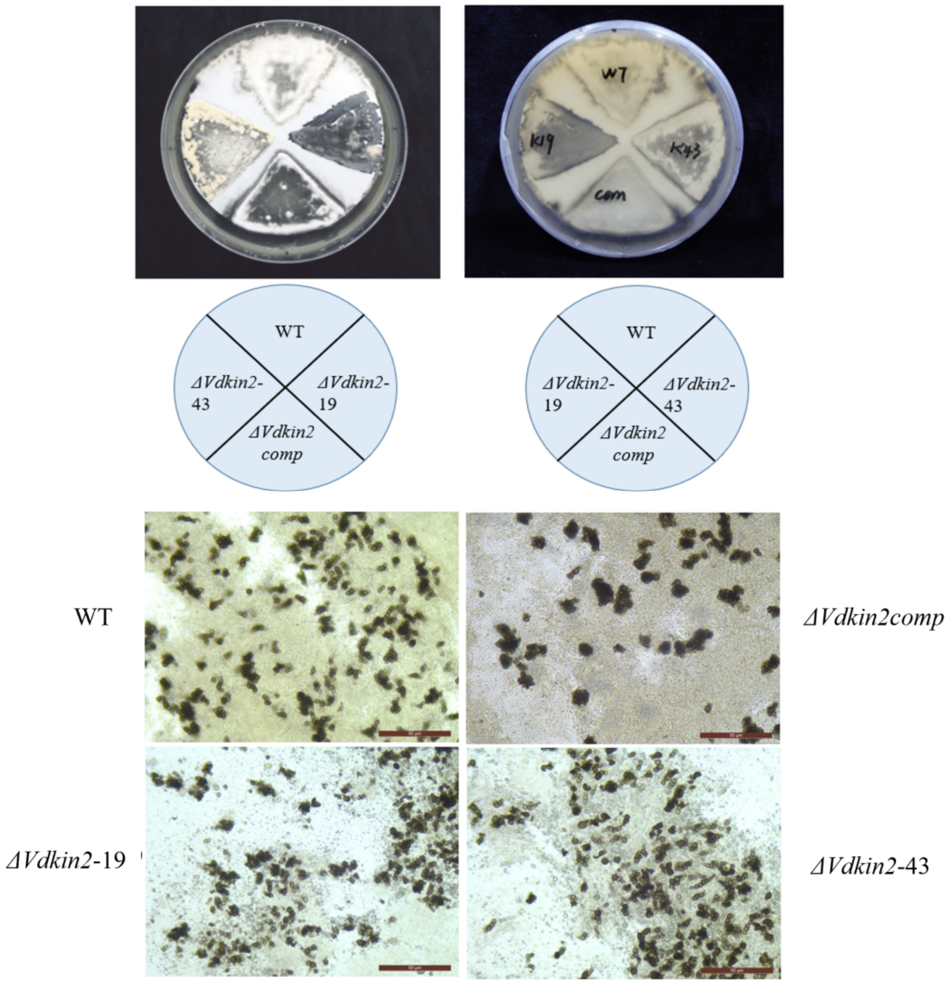

Figure S4. Strains were cultured on solid microsclerotia-inducing medium to induce microsclerotial formation. Mycelia and microsclerotia were scraped off the membrane for examination and counting under a microscope. Photos of the same Petri dishes were taken at 15 days post inoculation.

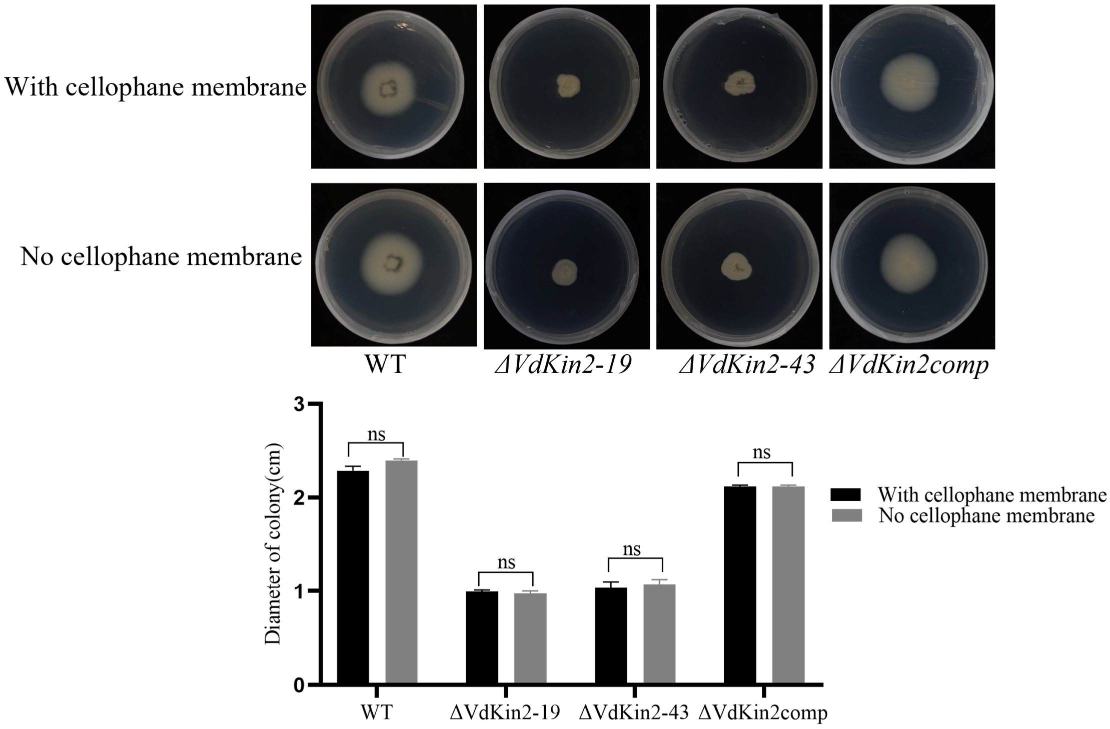

Figure S5. Strains were cultured on solid microsclerotia-inducing medium with or without cellophane membrane. The colonies were examined, and the diameter of each colony was measured at the 6th day.

Table S1. Primers used in this study.

Primer name	5′-3′	Use
5F	AGAAGAGTAATCTAGCAAACGTCGAAGCCTCGAAC	Amplifying 5' homologous arms
5R	TTAGTGAGGGTTAATTGCGCGCGCTAGACATGATGACGAC	Amplifying 5' homologous arms
3F	AGTTGTTCCCAGTGATCTTCGTGGAAGGCGAGATTCAGATG	Amplifying 3' homologous arms
3R	ATTACGAATTGGATCACGCGCTCGCAGCGGTTCTG	Amplifying 3' homologous arms
Hyg-F	GCGCAATTAACCCTCACTAAA	Amplifying Hygr cassette
Hyg-R	CGAAGATCACTGGGAACAACT	Amplifying Hygr cassette
5OUT	GACATACGACGTGGCAGCTG	Identify mutant
3OUT	CTGCTGAGCCCGGTTGTTCG	Identify mutant
Vdkin2comp f	CGGCCAGTGCCAAGCTTCAGTACCCGATAACGTCATG	Amplifying Vdkin2 complement
Vdkin2comp r	CAGTTAACGTCGAATTCCTCGCTATTGCCCGATGCTG	Amplifying Vdkin2 complement
18sRNAf	CCGCCGGTCCATCAGTCTCTCTGTTTATA	Identify mutant
18sRNAr	CGCCTGCGGGACTCCGATGCGAGCTGTAAC	Identify mutant
Probe F	CCGCGCTCCCGATTCCGGAAGTG	Southern blot probe
Probe R	GCTTCTGCGGGCGATTTGTGTAC	Southern blot probe
Vdkin2 exf	CGCCAACAGCATTAAGGTTG	Identify mutant
Vdkin2 exr	CTCGTCGTCGATGCTGGAAC	Identify mutant
G418Sep5F	CAATCTTCAAATCTAGAATGTCGTCTTCAGCTACTTT	Expressing *Sep5*
G418Sep5R	CCACCGCCTCCACTAGTTTTGCTGTCATTCTCGCCGT	Expressing *Sep5*
GFPF	TCTAGAGGATCCTTAATTAAATGGTGAGCAAGGGCGAGG	Colonization
GFPR	AGTTAACGTCGAATTCCTACTTGTACAGCTCGTCCATGCC	Colonization

Table S2. *Verticillium dahliae* strains used in this study.

Strain	Genotype description	Reference
V991	Wild type	Xie et al., 2017
ΔVdkin2	*Vdkin2* deletion mutant	This study
Vdkin2comp	*Vdkin2* complementary strain	This study
WT/GFP	Transformant of V991 expressing *GFP*	This study
ΔVdkin2/GFP	Transformant of ΔVdkin2 expressing *GFP*	This study
WT/VdSep5-GFP	Transformant of V991 expressing VdSep5-GFP	This study
ΔVdKin2/VdSep5-GFP	Transformant of ΔVdkin2 expressing VdSep5-GFP	This study

Supporting Information Methods

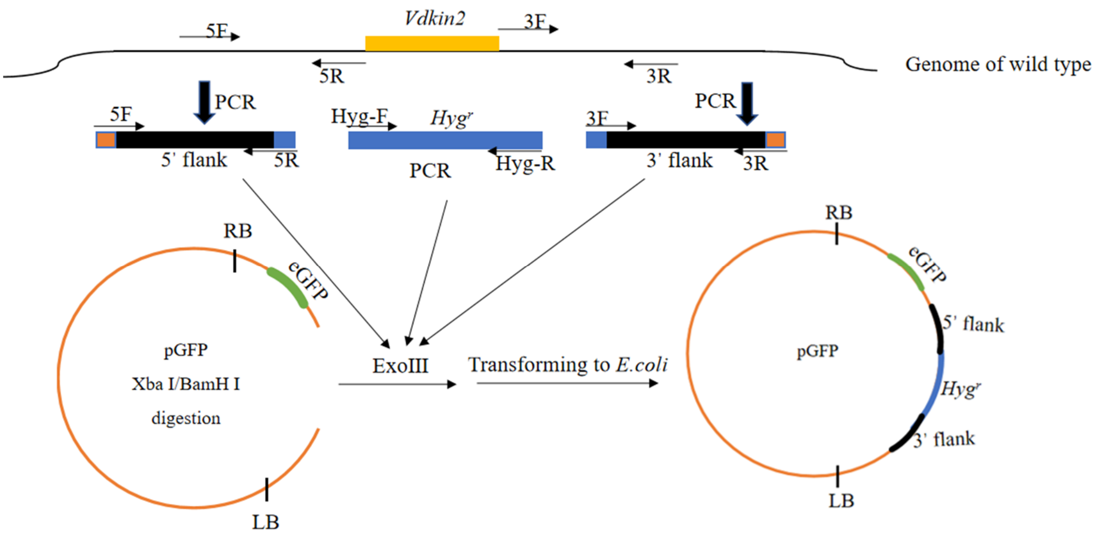

Construction of gene knock-out vectors.

1, The 5′ (5F/5R) and 3′ (3F/3R) flanking sequence and *Hyg^r* cassette (Hyg-f/Hyg-r) were amplified using high-fidelity enzymes.

2, The pGFP vector was digested using *Xba* I and *Bam*H I.

3, The digested pGFP vector, the 5′ (5F/5R) and 3′ (3F/3R) flanking sequence, *Hyg^r* cassette were mixed with ExoIII (TaKaRa Dalian Biotechnology) or Gibson Assembly Cloning Kit (NEB). *5F and 3R contain overhangs homologous to the pGFP vector, and 5R and 3F have overhangs homologous to the *Hyg^r* cassette , which allow for annealing.

4, The ligation mixture was transformed into *Escherichia* coli to generate gene knock-out vector.

5, The gene knock-out vector were introduced into *Agrobacterium tumefaciens* by electroporation.

6, ATMT of *V. dahliae* was performed as described previously (Xie et al. 2017 doi:10.1094/mpmi-01-17-0007-r).

7, The transformants were selected on PDA supplemented with cefotaxime at 500 μg/ml and hygromycin B at 50 μg/ml.

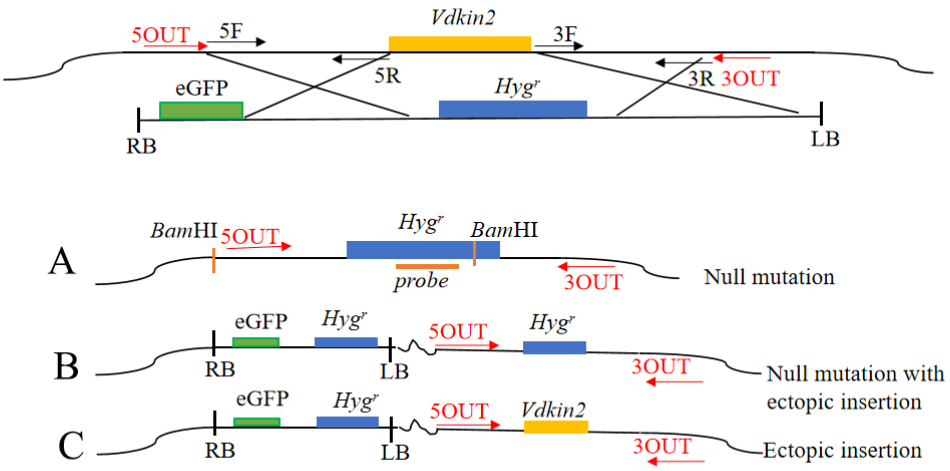

Identification of null mutant transformants.

1, Transformants are screened for GFP fluorescence under a microscope. The strains without GFP fluorescence were retained for further analyses. *Homologous recombination creates three types of transformants: **A:** null mutants, **B:** null mutants with ectopic insertions and **C:** ectopic insertion transformants. **B** and **C** have GFP fluorescence because the GFP gene was included in the T-DNA fragment (RB and LB). Null mutants (**A**) do have not GFP fluorescence because the GFP gene was eliminated in the null mutants.

2, Primers 5OUT and 3OUT are used for amplification gDNA from transformants. This length of null mutants should be longer than of WT or ectopic insertion transformants.

3, Reverse-transcription (RT)-PCR was performed to confirm the absence of *Vdkin2* mRNA in null mutants.

4, The gDNA were extracted with CTAB method (Kuhad, Kapoor et al. 2004 doi: 10.1007/BF02931383) and digested with *Bam*H I.

4, A *Hyg*r probe is used for southern blotting according to the protocol of the DIG High Prime DNA Labelling and Detection Starter Kit I (Roche, Mannheim, Germany)
